# Social Media Use Among Nonprofit Organizations in Rural Appalachia

**DOI:** 10.13023/jah.0102.05

**Published:** 2019-07-06

**Authors:** McKenzie Liegel, Jodi L. Southerland, Katie Baker

**Affiliations:** mliegel@wisc.edu; East Tennessee State University, southerlanjl@etsu.edu; East Tennessee State University, bakermk@etsu.edu

**Keywords:** Nonprofit organizations, social media use, Appalachia

## Abstract

**Introduction:**

Social media have changed the landscape of health communication for nonprofit organizations (NPOs). Yet, adoption and use of social media lag among NPOs in rural Appalachia due largely to limited infrastructure development.

**Methods:**

Semi-structured phone interviews were conducted in January–March 2018 with 21 NPO representatives in an 8-county region of rural Appalachian Tennessee. NPO representatives were asked questions pertaining to social media use and message content, effective communication strategies, and best practices in social media use. Transcripts were analyzed in April–May 2018 using thematic analysis.

**Results:**

The majority of NPOs had a Facebook page and recognized its promise as a communication tool. However, due to resource constraints, most NPOs used social media as a secondary communication strategy to complement traditional approaches. In terms of messaging, NPOs used social media primarily to share information and solicit donations or volunteers. Representatives identified several obstacles to social media use among NPOs in the region. These included limited organizational resources, community infrastructure, and household resources.

**Implications:**

Social media are inexpensive communication tools that NPOs in rural Appalachia can use to expand their digital footprint into hard-to-reach populations. Therefore, eliminating the digital divide across the U.S. is an important step toward enhancing rural NPOs’ capacity to serve their communities well. Opportunities for NPO staff to access low-cost professional development and training in the use of social media, specifically for social marketing purposes, are also essential.

## INTRODUCTION

Social media have changed the landscape of health communication for nonprofit organizations (NPOs). Over the past decade, broadband and wireless infrastructure have mushroomed throughout the U.S. resulting in nearly ubiquitous wireless coverage in most cities. Today nearly 90% of U.S. adults are online^1^ and seven-in-ten use social media.^2^ YouTube and Facebook are the most widely used platforms among U.S. adults, with more than half returning to these sites daily.^2^

Many NPOs have taken advantage of this shift in technology and use social media to communicate, share information, and engage in interactive feedback with their target audience.^3^,^4^ Not surprisingly, social media have several advantages over traditional forms of mass communication: cost-effectiveness, broader, rapid reach, ease of dissemination, and potential to build community identity and promote civic engagement.^5^

Nonprofit organizations that promote, protect, and improve the public’s health can take advantage of social media, as social media have the potential to allow for advocacy and engagement on important health-related issues,^6^ which, in turn, can help create a culture of health and improve health outcomes. This is particularly important in Appalachia which has experienced widespread and persistent poverty and poor health outcomes.^7^

Yet, adoption and use of social media lag among NPOs in rural Appalachia due largely to limited rural broadband infrastructure, limited organizational resources, and limited exposure to the potential impact of social media on organizational reach and communication efforts.^8^ For example, rural populations are less likely to use social media^2^ primarily because they have less access to reliable, affordable broadband services.^1^ Public perception of need for access to this technology may also be under-represented in rural communities because of remote location, sparse population, and higher median age compared with suburban and urban communities.^9^ Collectively, these factors have limited NPOs’ capacity to use social media effectively.

## METHODS

Purposive sampling techniques were used to recruit representatives from public health-related NPOs located in an 8-county contiguous region of Appalachian Northeast Tennessee. The IRS Exempt Organizations Select Check online search tool was used to identify NPOs who were located in this 8-county region. NPOs who engaged in initiatives to improve the public’s health were included in the study. Civic or other NPOs were excluded (e.g., PTO/PTA, athletic boosters, Little Leagues, VFW/Lodge Organizations). Recruitment methods included email invitations and telephone calls. Semi-structured phone interviews were conducted in January–March 2018 with representatives from 21 NPOs who agreed to participate in the study (42% overall participation rate). Representatives (e.g., CEO, Executive Director, President) who had knowledge of the communication strategies used by the NPO were interviewed. Consent was obtained before the audio-recorded sessions began.

Nonprofit organization representatives were asked questions pertaining to social media use and message content, effective communication strategies, and best practices in social media use. Interviews were recorded and transcribed verbatim. Transcripts were analyzed in April–May 2018 using Braun and Clarke’s (2006)^10^ thematic analysis. NVivo 12.0 was used to code the transcripts. The study was approved by the Institutional Review Board at East Tennessee State University (IRB# c0118.15sd).

## RESULTS

### NPO Characteristics

Tables 1 and 2 describe the characteristics of the NPOs. One-third (33.3%) of NPOs in the study had a multi-county service area and one-fourth (28.6%) were affiliated with a national organization (data not shown). The majority of NPOs (95.2%) had a Facebook page. However, only 42.8% of NPOs felt they had sufficient resources to use social media effectively and less than one-third (28.5%) had a social media marketing plan (Table 1). Among the most commonly used communication strategies, Facebook was cited as the most effective way for NPOs to communicate with their priority population, followed by direct communication (e.g., word of mouth, personal interactions, referrals, and monthly meetings). Print (e.g., newspaper and mailings), radio, and other forms of social media (e.g., Twitter and Instagram) were identified as the least effective methods (Table 2).

### Thematic Analysis

Results from the thematic analysis were organized into four main themes: use of social media as a secondary strategy, messaging, organizational capacity, and rurality (Table 3).

### Use of Social Media as a Secondary Strategy

The majority of NPOs used social media in some capacity to enhance their communication strategies but relied largely on traditional forms of communication to communicate with their priority population. For example, NPO representatives described using word of mouth and community relationships to spread their mission and disseminate information. When employed in health communication campaigns, Facebook was among the NPOs’ preferred social media platform, with other options such as Twitter and Instagram rarely in use. NPO representatives viewed social media primarily as a mechanism to extend the NPO’s reach to other communities and to bolster other communication efforts.

### Messaging

NPOs used social media mainly to share information and to solicit donations and volunteers. NPOs were far less likely to use social media as an advocacy or community mobilization tool or for creating user generated content. NPOs that engaged in more interactive messaging specific to programs, events, and initiatives via social media used live video streaming (e.g., Facebook Live), e-surveys, and hashtags. Live video streaming was used by NPOs to connect with followers who were unable to attend events in person. E-surveys were described as a tool to get input from the target audience to tailor programming. NPOs used hashtags to connect the audience with important themes within the broader community (e.g., #MeToo). Human interest stories also emerged as a prominent form of messaging among NPOs. Interestingly, NPOs used traditional media (e.g., newsletters and mailings) to share these stories to generate support (e.g., financial and volunteers) for their organization’s cause rather than disseminate the information on social media platforms.

### Organizational Capacity

Much of the discussion on organizational capacity centered on fiscal and personnel constraints. NPO representatives listed an array of challenges, which included lack of funds, staff, expertise, time, technology and equipment. Lack of dedicated staff with social media expertise and lack of time were the most commonly cited obstacles to social media use. Interviewees stated that staff wore many hats within the organization and were unable to spend sufficient time on social media efforts. In addition, many staff had limited experience using social media for professional purposes. Because of these challenges, many NPO representatives expressed a desire for professional development and training on social media use if funding were available.

### Rurality

Rurality also featured prominently in discussions with NPO representatives. Interviewees shared stories to highlight the community’s resourcefulness, shared identity, and strong social ties. NPOs have adapted their communication strategies to reflect the resources available to them. For many within the NPO’s service area, broadband and wireless services were unavailable, unreliable, or cost-prohibitive. In some cases, the most rural NPOs were either new to (≤6 mos) or not using their NPO’s Facebook page with any regularity. Thus, remote, rural NPOs relied almost solely on traditional forms of communication (e.g., word of mouth, relationship building, storytelling). For example, NPOs communicated event promotions or schedule changes via word of mouth at local businesses and churches, radio scanners (e.g., akin to police scanners that allow you to listen to two-way radio calls), and text messaging. In terms of obstacles, cultural norms and resource limitations featured prominently in discussions. Specifically, distrust of outsiders and limited interest in adopting new behaviors (e.g., new ways of communicating) were challenges faced by many NPOs. In addition, residents in low-wealth communities had high rates of residential mobility, which complicated NPOs’ attempts to reach them by phone or home address. Lack of community infrastructure (e.g., broadband and wireless access, social services) further complicated NPOs’ efforts to build strong connections with their priority population via targeted communication campaigns.

## IMPLICATIONS

In this qualitative study of NPO representatives in an 8-county region of rural Appalachian Tennessee, the analyses revealed that the majority of NPOs had a Facebook page and recognized its promise as a communication tool. Yet, resource constraints limited NPOs’ ability to use this platform effectively. As a result, most NPOs used social media as a secondary communication strategy to complement traditional approaches (e.g., mass media, word of mouth). In terms of messaging, NPOs used social media primarily to share information and solicit donations or volunteers. NPOs were less likely to use social media as an advocacy tool, to promote civic engagement or for creating user generated content. Representatives identified several obstacles to social media use among NPOs in the region. These included limited broadband infrastructure and household resources as well as limited organizational capacity, including a lack of social media skills and training among staff. Similar findings have been reported elsewhere.^3^,^4^,^8^

The present study provides a snapshot into NPOs’ social media use in rural Appalachia. The thematic analysis revealed that working within this cultural setting offers promise (e.g., strong sense of identity and rich social ties) juxtaposed with the need for resourcefulness (e.g., navigating resource limitations and remote locations). Eliminating the digital divide across the U.S. is an important step towards enhancing rural NPOs’ capacity to serve their communities well. For example, social media are inexpensive communication tools that NPOs in rural Appalachia can use to expand their digital footprint into hard-to-reach populations, including those who do not use the internet for health-related purposes^11^ and those who are reluctant adopters of this technology.^12^ It is important to note that broadband expansion alone will be insufficient for addressing health communication disparities among rural NPOs. Opportunities for NPO staff to access low-cost professional development and training in the use of social media, specifically for social marketing purposes, are also essential. To address cultural norms of distrust and hesitancy to adopt new communication tools, staff must be trained in targeting and tailoring health communication messaging to inform, engage and persuade their rural audiences while also leveraging the region’s traditions of storytelling and shared identity.

The study has several limitations. Subjective bias, particularly during data analysis, is a concern with qualitative research. Several strategies were employed to minimize bias and enhance the trustworthiness of the study including comparison of analysis by a skilled qualitative researcher, quantitative summary of key findings, and participant feedback to validate findings. Additionally, we did not collect participants’ demographic data nor data on NPO characteristics (e.g., size, mission, etc.). These data would have added much-needed context to our findings and subsequent implications and conclusions. Due to the nature of our sampling methodology, participation may have been limited to NPO representatives with the strongest interest in the topic. Lastly, the sample was comprised of senior level staff members (e.g., directors, CEOs, and presidents). Future studies should include mixed methodologies with a larger sample size, more diverse representation among NPO staff, and content analysis of the NPOs Facebook pages.

## CONCLUSION

Congress is working on making high-speed Internet more readily available to rural America. In the interim, NPOs in Appalachia will need to target communication modes available within their service area. NPOs and policymakers can use the findings in the present study to advocate for expansion of broadband and wireless services in rural Appalachia.

**SUMMARY BOX**

**What is already known about this topic?**

Social media have changed the landscape of health communication for non-profit organizations (NPOs). Yet, adoption and use of social media lag among NPOs in rural Appalachia.

**What is added by this report?**

Appalachian NPOs with limited resources have limited use of social media due to a host of environmental and socio-cultural factors.

**What are the implications for public health practice, policy, and research?**

Social media are inexpensive communication tools that NPOs in rural Appalachia can use to expand their digital footprint into hard-to-reach populations. Expanding broadband services and opportunities for low-cost professional development and training in the use of social media are important steps towards addressing health communication disparities among rural NPOs.

## Figures and Tables

**Table 1:** Social media use among NPOs in rural Appalachian Tennessee

	Yes N (%)	No N (%)
Does your organization have a Facebook page?	20 (95.2)	1 (4.7)
Does your organization have sufficient resources to use social media effectively?	9 (42.8)	12 (57.1)
Does your organization have a social media marketing plan?	6 (28.5)	15 (71.4)

**Table 2:** Most commonly cited communication methods among NPOs in rural Appalachian Tennessee

Communication Method	Most effective (%)	Least effective (%)
Facebook	57.1	4.5
Direct communication	33.3	4.5
Newspaper	9.5	19.0
Twitter	4.5	14.3
Instagram	4.5	14.3
Mailings	4.5	14.3
Radio	4.5	9.5

**Table 3:** Selected quotes from NPO representatives by theme

Theme	Participant Quote
Use of Social Media as a Secondary Strategy	But we also have our patients that we serve. They’re generally low income. Most of the time [*sic*] don’t have access to social media. We do still have a Facebook page and a Twitter account, and we do try to post on those, but generally most of our communication comes from phone calls or letters to our clients.
	We mobilized people through the traditional media of newsprint and TV and also radio. But also, we did mobilize them through social media. So, I think we used all areas of communication to get people involved.
Messaging	Well, we initially post [on Facebook] just to spread the word about our organization. Just what we do, who we are, what we’re trying to achieve. And then once the community kind of got an understanding of who we are, we post our schedule on there[Facebook]: where we’ll be throughout the region, what numbers you can call if you want to get signed up as a patient.
	Not necessarily on Facebook but when we do direct mailings for fundraising purposes, we use stories of kids because it’s the most compelling. Nobody really wants to hear about a company that needed us to help them. But the work we do with kids pulls at heart strings.
Organizational Capacity	We’re a very small staff and it’s hard for us to dedicate the time. I feel like we could do a lot more if we had funding for another staff member or students involved that could help us with that [social media]. I think it would be great if we could have some more financial resources or people that could do these things for us.
	We need so much. Obviously, I mentioned we could use at least one desktop and a couple of laptops. Really, it’s a lack of equipment.
Rurality	We were in a bind. We had to do it [campaign] and we had to come up with something fast. That’s where when you’re an organization like us in a rural area and you rely on word of mouth and a scanner, those are all quick things. They get out pretty fast. That’s when our situation becomes the advantageous situation.
	Peoples’ phone numbers and addresses change so much that we don’t rely [*sic*]. It would be a waste of money to send things out by mail and almost a waste of our time to sit and try and call the people. We do not rely on those kinds of things [phone numbers and addresses] in a poor area like we live in.
